# Cellular Pathology as Based upon Physiological and Pathological Histology

**Published:** 1861-05

**Authors:** 


### NORTH AMERICAN

### MEDICO-CHIRURGICAL REVIEW.

MAY, 1861.

### Analytical and Critical Reviews.

ART. I.—*Cellular Pathology as based upon Physiological and Pathological Histology.* By RUDOLF VIRCHOW. Translated from the German, by FRANK CHANCE, B.A., M.B. Illustrated by 144 Engravings on Wood. 8vo., pp. 511. London: Churchill, 1860.

THE reviewer of a new book may be compared, in his pursuit after scientific truth, to the miner who wanders about in search of gold. No matter with what notions he may enter upon his journey, it is seldom that he realizes his expectations. However closely he may scan his author, or follow him in his train of thought and of study, he but too often finds that the pages before him are a mere transcript, perhaps not even always happily disguised in language and style, of what he had repeatedly read before. If he now and then lights upon a little precious ore, it is generally so surrounded by rubbish as to require no little effort to bring it fully into view. Disappointment, if not utter disgust, is too frequently the only recompense which he obtains for his toil. We need not inform the reader that these remarks are not in anywise applicable to the treatise of Rudolf Virchow. Original in its design, and eminently so in its execution, it everywhere abounds in new facts, in plausible deductions, and in valuable suggestions, which stamp it as a great production.

The name of Virchow is not unknown to our readers. The pages of the Review have, on numerous occasions, borne evidence of the fruits of his vigorous intellect and of his varied and profound researches. Less than fifty years of age, he is one of the most indefatigable cultivators of medical science in the Old World, where he has established a reputation as brilliant as it is extensive and honorable. A native of Prussia, he rendered himself, early in life, on account of his radicalism in politics, offensive to his government; a feeling which was still further increased by his report on typhus and cretinism in Silesia, and which was probably the reason which induced him to quit Berlin and settle at Würzburg. In the latter city, so renowned for its university and its galaxy of distinguished men in the various branches of the arts and sciences, he was soon made a professor, and thus became associated with Scherer, Kölliker, and other medical luminaries, thirsting not less ardently than himself after knowledge and reputation. While here, zealously and laboriously occupied with the duties of his chair, he founded, in 1847, along with Remhardt, the Archives of Pathological Anatomy, and contributed numerous papers, all of them of a highly original cast, to the Würzburg Transactions. Many of these papers were speedily translated into foreign languages, and, together with most of his earlier pieces, were finally embodied in his "Collected Essays," published a few years ago in two stately octavo volumes, comprising his admirable researches on embolia, of which an account appeared in the May number of this Journal for 1858. He also projected, during his residence at Würzburg, the Cyclopædia of the Medical Sciences, he himself taking the department of pathology and therapeutics, upon which he has, however, published only the first volume.

A few years ago, a better feeling prevailing toward him on the part of the Prussian government, Virchow was recalled to Berlin, in the university of which he now occupies the chair of pathological anatomy, general pathology, and therapeutics. He is also Director of the Pathological Institute—an establishment which is indebted for its existence and reputation mainly to his influence—and physician to the Charité Hospital, one of the largest and best regulated receptacles of the kind in Germany. Of the vast industry of Professor Virchow, an idea may be formed when it is stated that he delivers not only full courses of lectures on the several branches of medicine confided to his care in the university, but that he passes daily eight or nine hours at the Charité, in prescribing for his cases and examining dead bodies, that he is constantly engaged upon new researches and the publication of important papers in his Archives of Pathological Anatomy, of which he still continues to be the editor, and that he makes it a business to read all the more important French, German, and English medical works as they are issued from the press. The translator of the Cellular Pathology states, in referring to this subject, as an additional proof of Virchow's industry, that he received from him, during the preparation of the English edition, at least fifty letters, most of them very long ones, explanatory of the text, and generally written within the first twenty-four hours after the requisition of Dr. Chance fell into his hands. With such an astonishing capacity for labor, and such an honesty of purpose to discharge well and truly all the various duties confided to him, it is not surprising that Rudolf Virchow should enjoy the very first rank among the medical philosophers of his own country, and that he should have achieved a most enviable reputation among foreign nations. It is said that his private practice is very restricted, and this we can readily believe, since his public avocations must of necessity absorb nearly all his time.

The present is the second edition of the Cellular Pathology, the first having appeared in 1858. Mr. Chance, the translator, has performed his task with great care and the strongest desire to interpret correctly the meaning of the distinguished author. His style, however, is, in consequence, occasionally too idiomatic, and devoid of that elegance so befitting a work destined to be so generally read. The wood-cuts are well executed, and greatly assist in the comprehension of the text. A French translation of the work was published at Paris in 1860.

The treatise before us is founded upon the course of lectures, twenty in number, delivered by Virchow in the Pathological Institute of the University of Berlin in the early part of 1858. The auditors were numerous, and consisted principally of medical men, for the most part practitioners of the city. The subjoined paragraph explains the motive which induced the author to prepare them.

"The object chiefly aimed at in them, illustrated as they were by as extensive a series of microscopical preparations as it was in my power to supply, was to furnish a clear and connected explanation of those facts upon which, according to my ideas, the theory of life must now be based, and out of which also the science of pathology has now to be constructed. They were more particularly intended as an attempt to offer, in a better arranged form than had hitherto been done, a view of the cellular nature of all vital processes, both physiological and pathological, animal and vegetable, so as distinctly to set forth what even the people have long been dimly conscious of, namely, the unity of life in all organized beings, in opposition to the one-sided humoral and neuristical (solidistic) tendencies which have been transmitted from the mythical days of antiquity to our own times, and at the same time to contrast with the equally one-sided interpretations of a grossly mechanical and chemical bias—the more delicate mechanism and chemistry of the cell."

In the Cellular Pathology, Virchow, as expressly avowed in the preface to the first edition of the work, assumes the attitude, not of a revolutionist, but of a reformer; he would preserve the old, and add the new. His desire is to combat false doctrine, and aid in clearing away the rubbish which incumbers the science and mars the fair proportions of the great temple of medicine. Conscious of having labored hard and zealously for the advancement of pathology, histology, and therapeutics, he feels that he is entitled to a fair hearing on the part of the public, and he therefore places with modesty, yet as one aware of his strength, the results of his researches and deductions into the hands of his professional brethren, asking them to give them a rigorous and impartial examination, rejecting what is false and embracing what is true. We are disposed, at the outset, to accord to Virchow the highest meed of praise as an original and discriminating observer; his work displays everywhere the traces of a philosophical mind, and an ardent desire to unravel truth and to extend the boundaries of our knowledge. In studying its pages one feels almost inclined to conclude that the gifted author has been sent on a prophet's errand to inaugurate a new era in medical science. His treatise has much in common with that of Paget, and it might, perhaps, be regarded as filling the same gap in medicine that that work does in surgery. The Cellular Pathology, however, is in advance of the production of the English author, and it bears a still stronger impress of originality, laying, as it certainly attempts to do, the foundation of a new pathology and therapeutics.

The cell-doctrine is of recent origin. Not many years ago, Schleiden, whose name is so intimately associated with the science of botany, enunciated the important fact that the ulterior structure of all vegetables consists of minute cells, closely aggregated together, more or less complex in their arrangement, and enjoying each a sort of independent life or separate individuality. Schwann, following closely in the footsteps of his countryman, confirmed the truth of these researches, and ere long a host of observers arose who contended for the existence of similar cells in animal bodies. According to this doctrine, which is now attracting so much attention, the cell is the ultimate morphological element of our tissues, the ultimate link in every structural chain in which there is any vital manifestation. Although intimately connected with other cells, it is, nevertheless, independent of its neighbor; it has its own life, its own territory, its own government, and it is by the conglomeration of such bodies that the various tissues and organs are formed. No growth or development can take place without a cell; a cell may perish, but its death does not necessarily involve the destruction of any other cell. As Harvey contended that everything originated from the egg, so, according to Virchow, everything has its rise in a cell.

"Where a cell arises, there a cell must have previously existed; *omnis cellula é cellulâ*, just as an animal can spring only from an animal, a plant only from a plant. In this manner, although there are still a few spots in the body where absolute demonstration has not yet been afforded, the principle is, nevertheless, established that, in the whole series of living things, whether they be entire plants or animal organisms, or essential constituents of the same, an eternal law of *continuous development* prevails."

Setting out with this idea of the existence of a cell and the possibility of its indefinite extension, or continuous proliferation, as he terms it, the author arranges the normal tissues into three categories, according to the natural disposition of the cells, or their relation with each other and the neighboring structures.

In the first group are included those tissues which consist exclusively of cells, the cells lying closely together, and thus composing what, in the modern sense of the term, forms the cellular texture. The second category comprises the tissues in which the cells are separated from each other by a certain amount of intermediate matter, technically called *intercellular*, which, however, at the same time serves to bind them together. To this division appertain what modern anatomists denominate the connective tissue, or what was formerly known as the cellular tissue. In the third class are embraced the nervous and muscular systems, the vessels, and the blood, those higher types of cell-forms and tissues which belong exclusively to the animal economy, although a few of them exhibit a transition to vegetable forms. This classification, of course, totally rejects the theories of elementary fibres and globules, formerly so much insisted upon by anatomists and physiologists, or, what is the same thing, it seeks to reduce the tissues to their primordial principles. The modern histologist thus speaks of elementary cells and tissues in the same manner as the chemist speaks of elementary bodies, only that the classification of the one is very simple, whereas that of the other is extremely complex.

Virchow lays great stress upon what he terms *cell-territories*, assigning to them important nutritive functions. He asserts that in those structures which are destitute of vessels distinct canals exist, whose business it is to distribute the nutritive juices through the intercellular substance, thus imparting to each atom of living matter its due share of nourishment. In speaking of the minute anatomy of tendon, the following remarks occur:

"You have here a fresh instance in support of my view in regard to cell-territories. I would parcel out the whole tendon, not into primary and secondary fasciculi, but rather into certain series of cells, connected in a retiform manner; to such series, moreover, I would assign a certain district of tissue, so that in a longitudinal section, for example, about half of each band of basis-substance would belong to one, the other half to another series of cells. What is, therefore, regarded as constituting the proper fasciculi of the tendon, would, according to this view, have really to be split up, and the tendon portioned out into a great number of nutritive districts."

This reticular arrangement is nowhere more clearly demonstrable than in the section of a tendon, and Virchow, therefore, regards this structure as a model type of cell-territories. It influences the extent of the districts invaded by disease; for "every disease which depends upon a disturbance of the internal disposition of the tissues, is always made up of the sum of the separate changes occurring in such territories."

Every cell in the body, whatever may be its character, form, situation, or structure, from the most humble to the most exalted, from the most simple to the most complex, possesses the power of attracting matters from the passing current of blood, whether in close contact with, or more or less remote from, it, of effecting within its cavity certain changes in these materials, and of returning these materials, thus transmuted or altered, to the circulation, appropriating them to its own nourishment and support, or throwing them off in the form of recrementitious or excrementitious substances.

The germ of the whole cellular pathology of Virchow seems to be comprised in the following paragraph. After having spoken of the functions and elective affinities of some of the higher cells, as those of the liver and kidney, he remarks:—

"Now I demand for cellular pathology nothing more than that this view, which must be admitted to be true in the case of the large secreting organs, be extended also to the smaller organs and smaller elements; and that, for example, an epidermis-cell, a lens-fibre or a cartilage-cell be, to a certain extent, admitted to possess the power of deriving from the vessels nearest to them, (not always indeed directly, but often by transmission from a distance,) in accordance with their several, special requirements, certain quantities of material; and again that, after they have taken this material up, they be held to be capable of subjecting it to further changes within themselves, and this in such a manner that they either derive therefrom new matter for their own development; or that the substances accumulate in their interior, without their reaping any immediate benefit from it; or finally that, after this imbibition of material, even decay may arise in their structure and their dissolution ensue. At all events, it seems necessary to me that great prominence should be assigned to this *specific action of the elements of tissues*, in opposition to the specific action of the vessels, and that in studying local processes we should principally devote ourselves to the investigation of processes of this nature."

Virchow is a warm advocate of the local origin of the *dyscrasiæ*, denying that they ever have their real seat in the blood. He totally dissents from the idea of the humoral pathologist, so prejudicial in its clinical bearings, that any change in the composition of the blood may be propagated *in this fluid itself*, and remain there as a permanent or persistent thing; not but that he believes that such a change may pertinaciously continue, or that it may be even transmitted from generation to generation. On the contrary, he regards the blood as a temporary tissue, constantly dependent upon other parts, and incapable of regenerating and propagating itself out of itself. To satisfy ourselves upon this point, it is only necessary to apply the same conclusions, which are universally admitted to be true, respecting the dependence of the blood upon the absorption of new nutritive matter from the alimentary canal to the various tissues themselves. Virchow, in further illustration of this subject, shall speak for himself:—

"When the drunkard's dyscrasia is spoken of, nobody of course imagines that every one who has once been drunk labors under a permanent alcoholic dyscrasia, but the common opinion is, that, when continually fresh quantities of alcohol are ingested, continually fresh changes also declare themselves in the blood, so that its altered state must continue as long as the supply of fresh noxious matters takes place, or as, in consequence of a previous supply, individual organs remain in a diseased condition. If no more alcohol be ingested, if the organs which had been injured by the previous indulgence in it be restored to their normal condition, there is no doubt but that the dyscrasia will therewith terminate. This example, applied to the history of all the remaining dyscrasiæ, elucidates in a very simple manner the proposition, that *every dyscrasia is dependent upon a permanent supply of noxious ingredients from certain sources.* As a continual ingestion of injurious articles of food is capable of producing a permanently faulty composition of the blood, in like manner persistent disease in a definite organ is able to furnish the blood with a continual supply of morbid materials."

The essential point, therefore, practically considered, the author continues, is to search for the local origin of the different dyscrasiæ, or to determine what organs and tissues are concerned in producing this derangement in the constitution of the blood, a task by no means always easy or successful.

The name of Virchow, as is known to the reader, is intimately associated with *leukæmia*, and it is, therefore, not surprising that the subject should have received a certain share of attention in these lectures.

Leukæmia, signifying white blood, consists essentially in an increase of the number of white globules, in consequence, generally, of a diseased condition of the spleen, or of this organ and of the lymphatic glands. In the healthy state the number of white corpuscles is comparatively small, there being, on an average, not more than one to about three hundred red ones; but in the affection under consideration the relative proportion is greatly augmented, being frequently as one to five, four, or even three. Indeed, in some of the worst cases that have been observed, the two kinds of globules existed nearly in equal numbers, thereby imparting to the whole blood, or, more properly speaking, to certain portions of it, as the clots found in the right cavities of the heart after death, a singularly white appearance, as if the fluid were intermixed with puriform particles. It was, no doubt, owing to this peculiar condition of the blood, that pathologists were so long kept in ignorance respecting the true nature of this disease, the colorless globules of the blood having, until a comparatively recent period, been mistaken for pus corpuscles. Its discovery seems to have been effected almost simultaneously by Virchow and

J. Hughes Bennett, of Edinburgh, the latter of whom has described it under the more appropriate name of leucocythemia, literally signifying white cell blood, a term universally adopted in England, France, and America. Dr. Bennett, in his claim to priority, to which, however, no allusion is made by the German author, has conclusively proved that his first case of leucocythemia was observed in March, 1845, and an account of it published in the following October, whereas Virchow's case did not occur until August of the same year; nor did he give publicity to it until the ensuing November.

When leucocythemia exists in its worst form, the blood, as it flows from a vein in the arm in venesection, occasionally exhibits whitish streaks, strongly denotive of the nature of the disease. Again, if blood thus taken be deprived of its fibrin by stirring, and allowed to stand, a voluntary separation speedily occurs, the two kinds of corpuscles sinking to the bottom of the receiver, where they form a double sediment, the lower stratum being red, the upper white, or of a puriform aspect. This occurrence is readily explained by the differences in the specific gravity of the two kinds of blood corpuscles, the red being much the heavier, and so reaching the bottom of the vessel sooner than the white.

Nearly all the reported cases of this disease have terminated fatally; one only is said to have been benefited by treatment, and that merely in a slight degree. Whether death is caused directly by this condition of the blood, or by the lesions which give rise to this wonderful increase of white globules, is still a mooted question. As the disease proceeds toward its close, a genuine hemorrhagic diathesis is often established, causing blood to issue from the nose and other mucous outlets, and giving rise to apoplectic effusions into the brain. The quantity of fibrin in leucocythemia may be normal, increased or diminished.

Virchow speaks of two varieties of this disease, the splenic and the lymphatic; but for such a distinction there is no just foundation, inasmuch as it is not always induced by enlargement of the spleen and lymphatic ganglions, but may be caused also by disease of the liver, thyroid gland, kidneys, supra-renal capsules, stomach and intestines, although in most instances the former structures are most seriously, if not exclusively implicated; and this circumstance would seem to lead to the inference that these structures exert a direct and positive influence upon the production of the white globules, which often exist in such abundance, and which impart to the malady its characteristic features.

*Pyæmia* claims attention in Lecture IX. Notwithstanding the labors bestowed upon it by different observers, the author thinks it "must still be reckoned among the most controvertible of subjects." One great, if not the principal, reason of this is, that, until recently, observers constantly mistook the white globules of the blood, often greatly increased in number in this disease, for pus corpuscles. The error thus committed naturally led to the supposition that the constituents of pus pre-existed in the blood, that it might be secreted by this fluid, and that it could also, like a simple liquid, find its way into the vessels, or, in other words, that pus, as such, could be reabsorbed. All these doctrines, at one time so prevalent and so specious, have become obsolete. *Pus, as pus, is never reabsorbed.* It is only the more attenuated portions of pus that can be thus disposed of, or find their way into the circulation. The mass of solid constituents, left behind when this process has been going on, is a *caput mortuum*, a mass destitute of vitality, containing the pus corpuscles in a shriveled condition, incapable of being further influenced by the absorbent vessels. It constitutes the concrete pus of the French, and the cheesy, inspissated product of other writers. Such pus material, which is by no means uncommon, may play an important part in the economy, ultimately undergoing softening, and so causing inflammation and ulceration, as secondary consequences. It is only in very rare cases that the broken-down matter is reabsorbed. When this concrete substance exists in the lungs, it may simulate certain forms of tubercle, from which, however, as will be shown further on, it completely differs. Healthy pus, as has long been known, is a very compound fluid, comprising a prodigious number of distinctive globules, of an essentially cellular nature. The following extract gives the author's views upon the subject:—

"Pus consists essentially of cells, which in their ordinary condition lie close to one another, and between which a small quantity of intercellular fluid (*pus serum*) exists. Within the pus corpuscles themselves lies a substance which is likewise provided with a great quantity of water; for nearly every specimen of pus, although it may look very thick when fresh, contains such a large amount of water that it loses a great deal more by evaporation than a corresponding quantity of blood. The latter only gives the impression of being more watery because it contains a great deal of free, (intercellular,) but relatively little intra-cellular, fluid, while in pus, on the contrary, there is a greater quantity of water in the cells, and less without them. When, then, reabsorption takes place, the greatest part of the intercellular fluid first disappears, and the pus corpuscles draw nearer to one another; soon, however, a part of the fluid from the cells themselves also vanishes, and in proportion as this is the case, they become smaller, more irregular, angular, and uneven, they assume the most singular forms, lie closely pressed together, refract the light more strongly on account of their containing a greater quantity of solid matter, and present a more homogeneous appearance."

Although, in general, only the fluid portions of pus are reabsorbed, there are cases, and these are the more favorable, where all the constituents of the mass, fluid as well as solid, find their way into the circulation; but, in order that such an event may take place, the pus must previously undergo a fatty metamorphosis, its cells being broken down, and replaced by an emulsive mass, a kind of milk, composed of water, albumen, fat, and salts:—

"These are the processes which may be denominated 'physiological reabsorption of pus;' a reabsorption, in which pus is not reabsorbed as such, but either only its fluid constituents, or its solid ones after they have been considerably altered by an internal transformation."

There is, however, one condition in which pus may find its way directly into the current of the circulation, as in the case of a wound, rendering a vessel more or less patulous. But this is not, properly speaking, a reabsorption, but an intravasation, as Virchow designates it. The same thing may happen where an abscess, lying close to a vein, and bursting through its walls, discharges its contents into its interior. Finally, another mode of transmission or of ingress is by means of the lymphatics running into a collection of matter. This mode of access, however, must be very uncommon, and it remains to be proved whether pus, as such, can really find its way at all into the blood through these channels, inasmuch as there is a strong probability that its corpuscles are always arrested in their onward course by the lymphatic ganglions. As a proof of the truth of this remark, Virchow refers to what happens in the process of tattooing, in which the skin, after having been pricked at numerous points by a needle, is rubbed over with a substance which is insoluble in the fluids of the body, as cinnabar or gunpowder, so as to leave a permanent stain. Of this substance some necessarily gets into the lymphatic vessels, and it has been ascertained that the larger particles of it, the size of which is less than that even of the smallest pus corpuscle, are always intercepted by the lymphatic ganglions, none of them passing into the internal organs. "Now when such molecules as these are unable to pass, when such extremely minute particles cause an obstruction, it would be somewhat bold to imagine that pus corpuscles, which are relatively large, could effect a passage." The lymphatic ganglions, however, are not to be considered as mere filters interposed between the different portions of the lymphatic vessels; on the contrary, it is highly probable that the fluids which are retained in them undergo some chemical change, whereby they are deprived, in great measure, of their noxious properties, so as to render them, upon their arrival in the blood, comparatively inoffensive.

We have not time to follow Virchow in all he says upon this subject. The subjoined paragraph comprises the summing up of his conclusions respecting it.

"Upon examining these different pathological processes in this manner one by one, it is really impossible to discover anything at all, which, in a morphological point of view, could even in a remote degree justify the assumption of a condition such as might be called pyæmia. In the extremly rare cases, in which pus breaks through into veins, purulent ingredients may, without doubt, be conveyed into the blood, but in such instances the introduction of pus occurs for the most part but once. The abscess empties itself, and if it be large, an extravasation of blood is more apt to ensue than the establishment of a persistent pyæmia. Perhaps we shall at some future time succeed, in the course of such a process, in discovering pus corpuscles with well-defined characters in the blood; at present, however, the matter stands thus, that it can most positively be maintained that nobody has hitherto succeeded in demonstrating, by arguments capable of supporting even gentle criticism, the existence of a morphological pyæmia. This name therefore must, as designating a definite change in the blood, be entirely abandoned."

It seems to us that the view taken of this question by Virchow is anything but philosophical. The word pyæmia signifies blood poisoning, but it does not follow when such poisoning takes place that pus, as such, should enter the circulation, especially not that we should be able to detect the presence of pus corpuscles in the blood during life or after death. Pus is often a very irritating fluid, and that it may contain poisonous matter is a fact familiar to every one. The virus of chancre and that of small-pox are in point. They both enter the circulation, and make an impression upon the system capable of enduring a long lifetime. It has already been seen that the more attenuated portions of pus may easily find their way into the system. Why may they not, then, become a source of contamination to the blood, altering its properties, rapidly impairing the vital powers, and leading to the formation of metastatic abscesses in various organs and tissues? To produce such a result it is not at all necessary that pus corpuscles should enter the circulation, much less that pus, as pus, should be reabsorbed. All the mischief may be done by the fluid constituents of the pus, and if we assume the possibility of such an occurrence, which even Virchow himself does not deny, for he distinctly recognizes it in another portion of the work under the appellation of ichorrhæmia, there is no valid reason, indeed no reason at all, why we should discard the word pyæmia, more especially as universal usage has sanctioned its employment.

The subject of *embolia* occupies a conspicuous place in the volume before us, being fully discussed in Lecture X. If Virchow's claims to priority as the discoverer of leukæmia may justly be disputed by Dr. Bennett, no such doubtful question meets us here. To the German alone belongs the field, and it is needless to say that it forms the basis of much of the reputation which he has so justly earned as a patient and careful observer. The subject, it is true, as will appear by a reference to the learned paper in the Review previously alluded to, had been incidently mentioned by other pathologists; cases had occasionally been published as exemplifications of the possibility of such an occurrence; but it remained for Virchow to study the entire question in its various bearings and relations, and to bring it for the first time prominently before the profession. The very name which he applied to it was until then unknown in science. His first paper was exhaustive, proving how powerfully he had applied himself to the investigation of the subject.

The formation of embolia is discussed along with other topics, as pyæmia and phlebitis, with which it is intimately associated. Cruveilhier and his school regarded the formation of clots in the veins as inseparably connected with inflammation. Virchow, without denying that inflammation may exercise such an influence, asserts that there may, on the one hand, be phlebitis independently of coagula, and, on the other, coagula without phlebitis. Restricting himself, therefore, to the consideration of the naked facts of the formation of clots, he proposes to comprehend the whole of the process under the name of *thrombus*, using it as a substitute for the different terms phlebitis, arteritis, and others of a like import, inasmuch as, to use his own expression, the affection essentially consists in an actual coagulation of the blood at a *certain fixed spot.*

"Upon investigating the history of these thrombi," says Virchow, "we find that the puriform mass which is met with in their interior does not originate in the wall, but is produced by a direct transformation of the central layers of the clots themselves, a transformation indeed which is of a chemical nature, and during which, with a result similar to that which can be artificially obtained by the slow digestion of coagulated fibrin, the fibrin breaks up into a finely granular substance and the whole mass becomes converted into *débris.* This is a kind of softening and retrograde metamorphosis of the organic substance, in the course of which from the very commencement a number of extremely minute particles become visible; the large threads of fibrin crumble into pieces, these again into smaller ones, and so on until after a certain time has elapsed the chief part of the mass is found to be composed of small, fine, pale granules. In cases in which the fibrin is comparatively very pure, we frequently see scarcely anything else than these granules."

There is, then, no pus, no purulent matter, but merely a puriform substance in these thrombi, the result of their disintegration. In addition to the granules above described, there are specimens which frequently contain other structures, especially cellular elements, of a rounded or angular shape, closely heaped together, and presenting one, two, or more nuclei, the whole bearing a very remarkable resemblance to pus corpuscles. Upon carefully following up the different stages of the process in the formation of thrombi, Virchow has been able to satisfy himself that these corpuscles are not adventitious, but that "they pre-exist, and that they do not arise within the clot, and are not forced into it. Even when quite recent thrombi are examined, the corpuscles are found in many places heaped up in great masses, so that when the fibrin breaks up, they are set free in such numbers that the débris are nearly as rich in cells as pus. It is with this process just as when water, which is thoroughly impregnated with solid particles, is frozen and then exposed to a higher temperature; when the ice melts, the inclosed particles must of course again come to light."

In all these processes there is, then, in the true sense of the term, no suppurative phlebitis, or, indeed, any phlebitis whatever, but merely a coagulation of blood, a clot, or thrombus, which finally breaks down and becomes disentegrated. How this thrombus is formed Virchow fails to inform us. He does not deny that phlebitis may not occur; but in this event the inflammation affects, he says, the walls of the veins, and not their contents, causing the former to become opaque and thickened by interstitial deposits, and sometimes even exciting suppuration or the formation of abscesses. He, however, admits that phlebitis may at times occasion thrombus, and in like manner arteritis and endocarditis, as when the inner surface of the lining membrane of the heart and vessels is marked by inequalities, elevations, depressions, and even ulcerations, thereby entangling the blood, and so favoring coagulation. "Still," he adds, "wherever phlebitis, in the usual sense of the word, takes place, the alteration in the coat of the vessel is almost always a secondary one, and indeed occurs at a comparatively late period."

Now if Virchow thinks that the blood ever coagulates in a vein, in an artery, or in the heart, as a physiological act, we entirely disagree with him; for we have never in all our dissections witnessed a result so utterly at variance with the healthy circulation. We regard the thing as simply impossible. The only way in which we can account for the formation of clots or thrombi, during life, is to suppose that they are the result of a local, circumscribed, or diffused inflammation, forming so many points, roughened with plastic material, to which the fibrin of the blood, as it sweeps along, becomes attached, and so conduces to coagulation or the development of thrombi. We know of no other theory adequate to explain the occurrence.

The manner in which these clot-formations are broken down and ultimately removed is thus described by the author.

"The process runs its course in such a way that the most recent parts of the thrombus always consist of the blood which has most lately coagulated. The softening, the partial liquefaction, generally commences in the centre, in the oldest layers, so that, when the thrombus has attained a certain size, there exists in the midst of it a cavity of larger or smaller dimensions, which gradually enlarges and keeps approaching more and more closely to the wall of the vessel. But in general this cavity is shut off in an upward and downward direction by means of a more recent and tougher portion of the clot which, after the manner of a cap, takes care that, as Cruveilhier expresses himself, the 'pus' remains sequestered, and all contact between the *débris* and the circulating blood is prevented. Only sideways does the softening extend until it at last reaches the wall of the vessel itself; this becomes altered, it begins to grow thicker and at the same time cloudy, and ultimately even suppuration takes place within the walls."

There are two ways in which these clots may produce serious secondary effects; by the admixture, namely, of the dissolved matter with the blood, and by the detachment from the softened thrombus of various sized fragments which are afterward propelled along with the blood and forced into remote vessels, acting thus obstructingly, if not fatally, to part and even to system. Thus, in the brain they may cause apoplexy, in the heart an arrest of the circulation, in the lungs asphyxia; while in the smaller vessels they may provoke inflammation, dropsy, atrophy, dry gangrene, and other lesions. These disintegrated and softened masses, so pernicious in their tendency, constitute, it may now be observed, the so-called embolia of Virchow. Their study deserves the careful consideration of every pathologist and practitioner.

There is a condition of the blood to which the term *melanæmia* has been applied, in reference to the colored elements which are sometimes met with in this fluid. The affection, which appears to be intimately allied to leukæmia, has attracted the notice of various observers, as Stiebel, Meckel, Frerichs, and Tigri, who have all published cases illustrative of its character. Virchow himself has seen only one instance of it, and he therefore acknowledges his inability to throw any material light upon its nature, or to determine what influence, if any, it exerts upon the state of the blood, especially as to its contaminating power. Their development would seem to be in some way connected with enlargement or disease of the spleen, as in all of the reported cases of the affection this organ was found to be very unhealthy.

*Melanic* blood corpuscles occasionally occur in large quantities. They exist very sparingly in healthy individuals, although Schultz, who has devoted much attention to their study, declares that they are always present in considerable numbers. They have thus far been met with in greatest abundance in intermittent and typhoid fever, in cyanosis after cardiac disease, in epidemic affections, and in blood poisoning consequent upon severe accidents and operations. Virchow agrees with Schultz that these changes in the color of the blood corpuscles are due to a species of decay, a kind of *moulting* of the blood, or preparation for the really excrementitious transformations.

A large space is devoted to the discussion of the *fatty degeneration;* but, although the subject is replete with interest, especially when treated by a man of the vast scientific knowledge and skill of Virchow, there are so many other points to be examined that we shall content ourselves with a few brief extracts.

"We have now compared a series of examples of fatty degeneration, and may henceforth confine ourselves to the consideration of genuine *fatty metamorphosis,* in which the normal structure of the part is ultimately destroyed, and the place of the histological elements is gradually occupied by a purely emulsive mass, or, more concisely, *fatty débris.* It makes no difference whether it is a pus cell, a connective tissue corpuscle, a nerve or muscular fibre, or a vessel which experiences the change; the result is always the same; namely, milky débris, an amorphous accumulation of fatty particles in a more or less highly albuminous fluid. But though we hold to the agreement of all cases of fatty metamorphosis in this respect, it by no means however follows, that the importance of this change as a morbid process is in every case the same. This you may at once infer from the circumstance that, while I have introduced this process to your notice in the category of purely passive disturbances, one of the very structures which we most frequently find in it, the granule globule, has been regarded as a specific element of inflammation. For years an inflammatory globule [exudation corpuscle] was looked upon as an essential phenomenon in the process of inflammation, and in fact the frequency with which cells in a state of fatty degeneration are found in inflamed parts affords sufficient proof that, in the course of inflammatory processes, which it is impossible we should ever regard as simply passive processes, such transformations must take place."

To the above remarks, it is proper to add that the author considers the early stages of fatty degeneration to be nearly always preceded by a *cloudy swelling,* as he terms it, occasioned by the deposition of a large quantity of matter by which the parts are increased in extent and density. He does not admit that these phenomena are due to the superaddition of exudation proceeding from the vessels, for similar changes, he says, occur in parts where no vessels exist. It is only when the accumulation has attained such dimensions as to endanger the natural constitution of the elements that a fatty disintegration is set up in their interior. Moreover, he declares that, although there is, using the expression in a general sense, an inflammatory form of fatty degeneration, yet, strictly speaking, this form is only a late stage of the process, a kind of termination, announcing the commencement of the disintegration of the tissue, when the part deprived of the power of maintaining a separate existence is abandoned in some degree to the play of chemical affinities, or, in other terms, when it is almost ready to perish. When the essential elements of a tissue have been thus changed there cannot afterward be any restoration of its normal structure. Hence this form of fatty degeneration is of great practical consequence. The following remarks are worthy of attention.

"When inflammation takes place in a muscle and in its course the primitive muscular fasciculi fall into a state of fatty degeneration, as a rule they also perish, and we afterward find a loss of substance in the muscle at the spot where the degeneration took place. The kidney, whose epithelium has passed into a state of fatty degeneration, nearly always shrivels up, and the result is a permanent atrophy. In exceptional cases something perhaps occurs, which reminds us of a regeneration of the epithelium, but usually a collapse of the entire structure ensues. The same thing is witnessed in the brain in yellow softening, no matter how it may have been caused. Whether there have been inflammation or not, a vacuity is formed, which is never again filled up with nervous matter. Perhaps a simple fluid may replace the wanting tissues, but as to any reproduction of a new, functionally active part, that must ever be out of the question."

The *amyloid degeneration* is discussed in Lecture XVII. Until described by Virchow, about ten years ago, its true nature was concealed under what used to be known as the lardaceous or waxy alterations. Indeed, up to this period, it had also not unfrequently been taken for a peculiar fatty matter, albumen, fibrin, or colloid. The rudiments of the discovery were furnished by the application of iodine to the nervous centres, the reaction of which disclosed the existence of the so-called "corpora amylacea." Soon afterward it was detected by Virchow and Meckel in other organs, so that there are, at the present moment, hardly any structures in which it has not been observed. Thus, it has been found in the heart, liver, spleen, kidneys, uterus, the lymphatic ganglions, the minute branches of the arteries, the interior of cartilages, the whole alimentary track, in the mucous membrane of the urinary apparatus, and in the prostate gland.

The amyloid matter presents itself in two forms—as a free substance adherent to the outside of the tissues, or lying, as the author expresses it, between their elements, and as an infiltration of the tissues, in which all their constituents, parenchymatous and interstitial, are filled with it. In the former variety, the matter consists of bodies which, in their chemical properties, are more analogous to real vegetable starch; their form, too, bears a remarkable resemblance to vegetable starch granules, being more or less round, or oval, and arranged in a succession of concentric layers. Their volume is sometimes very large, and they are always particularly distinct in the "corpora amylacea" of the nervous system, in the prostate gland of old people, and in certain conditions of the lungs. Occasionally a number of these bodies are piled together, and then they may assume a "gigantic" size, having perhaps a diameter of two lines, so that they can easily be separated from the tissues in which they are imbedded, and examined with the naked eye. Under the action of iodine, they exhibit, like vegetable starch, a blue color, or a green color, if they are mixed up with much albuminous or nitrogenous matter.

In the other variety of amyloid degeneration, the mode of infiltration bears a very close resemblance to that of lime in calcification of the arteries; and the change differs still further from the preceding, in the fact that the new substance never becomes blue under the influence of iodine alone, at least no case of the kind has yet been observed. On the contrary, the contact of the fluid produces a peculiar yellowish-red color, and a perfectly blue or violet color only when the application of iodine is followed by the cautious addition of sulphuric acid.

The source of the amyloid substance is not determined. Virchow has sought for it in the blood, but in vain. However this may be, the affection to which it gives rise already occupies a high position among pathological processes. It has been shown that organs which are its seat are eventually entirely deprived of their functions; the glands, for example, being incapable of secreting their appropriate fluids, and the vessels of carrying nutritive matter to the tissues through which they are distributed. In the bowels, this metamorphosis induces diarrhœa; in the kidneys, dropsy and other symptoms of Bright's disease; in the liver, an arrest of the biliary secretion. In the advanced stages of the disease, the patient falls into a state of excessive marasmus, and finally dies exhausted, in many cases from sheer starvation.

In treating of *inflammation*, Virchow lays down his peculiar notions respecting the intimate nature of this process; and so important do we deem these views, that we shall endeavor to give a brief abstract of them for the benefit of the reader. It will be perceived that they are altogether of a revolutionary character.

Virchow sets out with the assertion that inflammation is not a real entity, or a process everywhere identical in its character, as has been so often supposed, but a process essentially similar to other morbid actions, differing, in fact, from them only in its form and course. He believes that irritation must be taken as the starting-point in the consideration of inflammation, for it is impossible to conceive of such an occurrence without the application of some hurtful stimulus. The principal sources of this irritation are three—the functional, nutritive, and formative—the first playing, as he imagines, the least important part in the process:—

"If, therefore, we speak of an inflammatory stimulus, we cannot properly intend to attach any other meaning to it than that, in consequence of some cause or other external to the part which falls into a state of irritation, and acting upon it either directly or through the medium of the blood, the composition and constitution of this part undergo alterations which at the same time alter its relations to the neighboring parts, (whether they be blood-vessels or other structures,) and enable it to attract to itself and absorb from them a larger quantity of matter than usual, and to transform it according to circumstances. Every form of inflammation with which we are acquainted may be naturally explained in this way. With regard to every one, it may be assumed that it begins as an inflammation from the moment that this increased absorption of matters into the tissue takes place, and the further transformation of these matters commences."

He discards the idea that hyperæmia, or vascular turgescence, is present at the commencement of inflammation, alleging that, in those parts, as the cornea, the cartilages, and tendons, in which no vessels exist, the changes produced by this morbid process are in no respect different from what they are in the vascular structures.

The deposit of fibrin, which all pathologists have heretofore regarded as a direct product of the vessels of the affected structures, Virchow insists upon is not an inflammatory exudation at all, but merely the material generated by the inflamed parts, through a change in their condition. The subjoined passages will serve to explain more fully his views upon the subject:—

"While until now fibrin has been regarded as a real transudation from the liquor sanguinis, as the outflowing plasma, I have proposed the explanation, that the fibrin, like the mucus, is a local product of those tissues, on and in which it is found, and that it is conveyed to the surface in the same way as the mucus of the mucous membrane. I then showed you, how we have in this way a most ready explanation of the fact, that in proportion as, in a given tissue, the production of fibrin increases, so also the amount of fibrin in the blood increases; and that the fibrinous crasis is just as much a product of the local disease, as the fibrinous exudation is a product of the local metamorphosis of matter. Never has any one—any more than it is possible for him by a change of pressure directly to produce mucus from the blood in any place which does not itself produce mucus—been able to produce fibrin by any change in the pressure of the blood; what transudes never consists of anything but serous fluids.

"I am accordingly of opinion that, *in the sense* in which it has usually been assumed to exist, *there is no inflammatory exudation at all*, but that the exudation which we meet with is essentially composed of the material which has been generated in the inflamed part itself through the change in its condition, and of the transuded fluid derived from the vessels. If, therefore, a part possesses a great number of vessels, and particularly if they are superficial, it will be able to furnish an exudation, since the fluid which transudes from the blood conveys the special products of the tissue along with it to the surface. If this is not the case, there will be no exudation, but the whole process will be limited to the occurrence in the real substance of the tissue of the special changes which have been induced by the inflammatory stimulus.

"In this manner, two forms of inflammation can be separated from one another; the *purely parenchymatous inflammation*, where the process runs its course in the interior of the tissue, without our being able to detect the presence of any free fluid which has escaped from the blood; and the *secretory (exuda-*

*tive*) *inflammation*, (which belongs more to the superficial organs,) where an increased escape of fluid takes place from the blood and conveys the peculiar parenchymatous matters along with it to the surface of the organs. That there are two different forms is clearly shown by the fact that they occur, for the most part, in different organs. There are certain organs which, under all circumstances, only suffer from the parenchymatous affection, and others again which in nearly every instance exhibit a superficial exudative inflammation."

We offer no comments upon these views other than a mere reference to the palpable contradiction contained in the statement that, "*while in this disease there is no inflammatory exudation at all, the exudation which we meet with is essentially composed of the material which has been generated in the inflamed part itself, through the change in its condition, and of the transuded fluid derived from the vessels.*" We confess our inability to interpret the learned author's meaning.

The last lecture in the book is devoted to the consideration of the nature of *new morbid formations*, embracing the various classes of cancer and tubercle, subjects which have engaged much of the author's attention, and upon which he has apparently thrown new light.

All these formations have their origin in connective tissue, "or in parts equivalent to this tissue;" their elementary principles are nearly all of the same nature, and their similarity is still further shown by the division and multiplication of the nuclei and cells. These traits are found alike in the benign and malignant formations, in the hyperplastic and heteroplastic. They are, however, altogether of a transitory nature; or, in other words, they appertain only to the earlier periods of these growths, for by degrees they individualize themselves, each structure exhibiting some characteristic feature by which it may subsequently be distinctly recognized.

We shall not follow Virchow in his remarks upon the faulty nomenclature of these formations—a subject which has caused so much lamentation among pathologists during the last half century, and been such a stumbling-block to practitioners—nor shall we attempt to present an analysis of all that he says respecting individual formations. It will suffice if we take up a few of them.

*Tubercle* the author defines to be a granule, or a knob, consisting of a new formation, which has its origin generally in connective tissue, and which, from the time of its earliest development, is necessarily of a cellular nature. The characteristic feature of this new product is the extraordinary abundance of nuclei, as is shown by the fact that, if it be examined as it lies imbedded in the substance which invests it, it appears to be composed almost exclusively of these bodies. A more thorough exploration, however, soon proves that this is an error. By isolating the constituents of the mass, distinct cells are discernible, some of which are very small, and provided each with only one nucleus, and that of extreme exiguity, while others are much larger, and contain from a dozen to twenty-four or even thirty nuclei. In the latter case, the nuclei are always very diminutive, homogeneous, and shining in appearance.

The subjoined extract will serve to show the opinion which Virchow entertains respecting the relations which tubercles sustain to other morbid products, as pus and cancer, the nature of its organization, and its liability to various degenerations.

"This structure, which in its development is comparatively most nearly related to pus, inasmuch as it has the smallest nuclei and relatively the smallest cells, is distinguished from all the more highly organized forms of cancer, cancroid and sarcoma, by the circumstance that these contain large, voluminous, nay, often gigantic corpuscles with highly developed nuclei and nucleoli. Tubercle, on the contrary, is always a pitiful production, a new formation from its very outset miserable. From its very commencement it is, like other new formations, not unfrequently pervaded by vessels, but when it enlarges, its many little cells throng so closely together, that the vessels gradually become completely impervious and only the larger ones, which merely traverse the tubercle, remain intact. Generally fatty degeneration sets in very early in the centre of the knot, (granule,) where the oldest cells lie, but usually does not become complete. Then every trace of fluid disappears, the corpuscles begin to shrivel, the centre becomes yellow and opaque, and a yellowish spot is seen in the middle of the gray translucent granule. This is the commencement of the *cheesy metamorphosis* which subsequently characterizes the tubercle. This change advances from cell to cell farther and farther outward, and it not unfrequently happens that the whole granule is gradually involved in it."

Again, referring to the transformation of tubercle, some distance farther on, Virchow says:—

"As soon as it has once attained a certain size, as soon as the generations of new corpuscles, which develop themselves out of the old histological elements by a continual succession of divisions, at last lie so close to one another as to cause a mutual arrest of development, gradually to induce the disappearance of the vessels of the tubercle, and thereby to cut off their own supplies, then they begin to break up, they die away, and nothing remains behind but débris—shrunken, disintegrated, cheesy material."

There is here, it will be perceived, a distinct recognition of the existence of vessels in tubercle, and the assertion, emanating as it does from one of the acknowledged microscopical leaders of the day, cannot fail to carry conviction into the minds of the most skeptical. For nearly a quarter of a century we have ourselves strenuously contended in our lectures and in our writings, in season and out of season, for the presence of this essential element in tubercle; for we could not, by any facts, reason, or argument that we could call to our aid, account for the various changes and transformations which tubercle is liable to undergo in any other manner. The only reward which we received for our constancy to our opinion was ridicule, if not down-right obloquy and contempt; sometimes a kind of pity for one so blind and obstinate! But now that Virchow is in the field, teaching the cellular origin and nature of tubercle, its power of proliferation, its similarity to all other new formations, and the existence of vessels in it, all doubt must necessarily cease. "*Magna est veritas, et prævalebit.*"

Another doctrine which is distinctly enunciated in this work is the inflammatory origin of tubercle. Here, again, there is no special novelty, for this doctrine has been strenuously taught in this and other countries for a long time. Latterly, however, many have abandoned their ground, and espoused the opposite side of the question, owing chiefly, it would seem, to the influence exerted upon the professional mind by the advocates of the stimulant practice in the treatment of disease, of which John Brown was in the last century, as Dr. Todd has been in this, the great prototype. There are, however, pathologists who have held on firmly to their views, despite the changes going on around them; men who do not barter away their opinion merely for opinion's sake.

Upon the minute structure of *cancerous* formations, Virchow has not favored us with anything of a very satisfactory nature. Scirrhus hardly meets with any allusion, and encephaloid fares but little better. The fact is, the whole subject is so jumbled up as to render it extremely difficult to catch the author's meaning.

The *colloid* growth, first described by Laennec in the early part of the present century, he proposes to call the mucous cancer, from the fact that there is always a certain amount of mucus contained in its intercellular substance. He finds its histological type in the normal structure of the umbilical cord. By this change of nomenclature, we know, says Virchow, exactly what is before us; we know it is cancer, the stroma of which differs from that of ordinary cancer in the presence of mucus and the gelatinous nature of the mass.

The term *epithelioma*, recently introduced by Hannover, the author condemns as being wholly inadmissible in the form of tumor which it was originally designed to designate, inasmuch as epithelial cells are also found in other morbid growths, both benign and malignant. We quote the following paragraph in illustration of his opinion upon this interesting point. Our own view has always been, since the term epithelioma has come into vogue, that the disease which it is intended to characterize is in general merely a form of scirrhus, or scirrho-encephaloma, modified in its structure by the nature of the tissues in which it is developed.

"Again, if you attempt to distinguish cancroid growths from real cancer by the epithelial structure of their elements, you will herein too give yourselves trouble in vain. Cancer proper has also elements of an epithelial character, and you need only turn to those parts of the body, where the epithelial cells are irregularly developed, as for example, in the urinary passages, and you will meet with the same curious bodies, provided with large nuclei and nucleoli, which are described as the specific, polymorphous cells of cancer. Cancer, cancroid, or epithelioma, pearly tumors or cholesteatoma, nay, perhaps the dermoid growths which produce hairs, teeth, and sebaceous glands, and so frequently occur in the ovary—all these are formations in which there is a pathological production of epithelial cells, but they constitute a graduated series of different kinds, which extend from those which are entirely local, and, in the usual meaning of the word, perfectly benignant, to the extremest malignity. The mere form of the cells which compose a structure is of no decisive value. Cancer is not malignant because it contains heterologous cells, nor cancroid benignant because its cells are homologous—they are both malignant, and their malignity only differs in degree."

Virchow teaches that the malignancy of morbid growths is materially influenced by the amount of juice pervading them. Those for example which are dry are comparatively benignant, while those which are moist or succulent are always more or less malignant. The former, in consequence of this peculiar attribute, are less liable to spread; or, what is the same thing, their influence is chiefly local, and they often remain a long time without producing any serious mischief. "In cancer proper the local progress is often very rapid and the disease early becomes general; a cure, even for a short time, is so rare that in France the complete incurability of cancer, properly so called, has been asserted and maintained with success."

The opinion that ordinary cancer is incurable by medicine or the knife, is, we presume, not limited to France. It pervades the entire educated and experienced portion of the profession in all parts of the world. We have, ourselves, in an extensive practice of upwards of twenty-five years, involving numerous operations for the cure of carcinoma in various parts of the body, not seen a solitary instance that was eradicated by medicine, or that did not recur sooner or later after extirpation, and ultimately carry off the patient.

But we must bring this notice to a close. In doing so we can conscientiously declare that, although our task has by no means been a light one—owing to the twofold reason that a number of topics have been confusedly mixed up with each other, when they ought to have been discussed separately, and to our anxiety to present a clear and satisfactory outline of some of the more important pathological deductions in which the work abounds—we part with the author with feelings of deep regret.

In traveling with him over his interesting and beautifully illustrated pages, we were constantly impressed with the conviction that we were in the company of a great man, an honest cultivator of pathology, intent upon the acquisition of new medical territory by the development of new scientific truths. It would, perhaps, be premature to pronounce a definitive judgment upon the work which we have thus rapidly passed in review, or to form a final decision respecting the real value of the labors and researches of Virchow. The readers of the Cellular Pathology will regard these matters very much according to the stand-point from which they may survey them, or according to their previous notions of pathology, and their ability or inability to interpret correctly the author's language and doctrines. To appreciate the work, and to do full justice to its teachings, it must be thoroughly dissected, carefully analyzed, and profoundly studied. A superficial perusal must inevitably eventuate in disappointment. Whatever the ultimate verdict of the profession may be in regard to it, it is easy to foresee that it must exert, at least for a time, an extraordinary influence upon the medical mind of this and other countries. One effect, and not the least important, that may be expected from its publication, is that it will lead to new inquiries and investigations, whereby the great truths which Virchow has disclosed will be placed in a clearer light and clothed in a more enduring garb, while the errors, if any there be, will be exposed and corrected. Already a number of his statements are received and cherished as established facts in science; stamped with the great seal of the profession both in the Old World and the New. That all that he has written in this book is true, or capable of withstanding the test of varied, patient, and elaborate scrutiny, no one, not even the author himself, will pretend. Amid so much good grain there must necessarily be some chaff.

It has been said of Virchow that he is an enthusiast, and that he is ambitious of becoming the founder of a new school, the school of cellular pathology. All seem willing to admit that he possesses a high order of talent, blended with unquestionable genius, extraordinary industry, and a boundless zeal in scientific pursuits. It is asserted that he lacks judgment, that he is hasty in his generalizations, and that his statements are often contradictory, and at times even absurd. We bring no such charge against Rudolf Virchow. If his love of science makes him an enthusiast, and if his ambition impels him to the performance of labors calculated to throw new light upon obscure points in pathology and therapeutics, are we to arraign him for that? If a tithe of what he has written in this book be true, then must we unquestionably and without hesitation regard Virchow as an able expounder of science, an original investigator, an admirable observer, a great thinker. Time determines all things, and to this arbiter the illustrious German may well submit his claims to the gratitude of this and of future ages. We should neither adopt hastily nor reject foolishly. The Cellular Pathology furnishes a subject on which every sensible inquirer may prove his ingenuity. "*Materiem, qua sis ingeniosus, habes.*" We have here ourselves merely glanced at it; to do it full justice would require a much larger space than we can afford. We have opened the casket, but taken out a few only of the jewels.

